# 非小细胞肺癌治疗的新靶点：*EML4-ALK*融合基因

**DOI:** 10.3779/j.issn.1009-3419.2011.06.11

**Published:** 2011-06-20

**Authors:** 慧娟 王

**Affiliations:** 450003 郑州，河南省肿瘤医院内科 Department of Medical, Henan Tumor Hospital, Zhengzhou 450003, China

**Keywords:** EML4-ALK融合基因, 间变性淋巴瘤激酶, 肺肿瘤, EML4-ALK, Anaplastic lymphoma kinase, Lung neoplasms

## Abstract

3年前棘皮动物微管相关类蛋白4（echinoderm microtubule-associated protein-like4, EML4）与间变性淋巴瘤激酶（anaplastic lymphoma kinase, ALK）融合基因被发现存在于部分非小细胞肺癌（non-small cell lung cancer, NSCLC）中。该融合基因常见于不吸烟的肺腺癌患者，有其独特的病理学特征，可以诱导肿瘤生成。ALK抑制剂能够作用于该基因的下游信号传导通路并拮抗其促肿瘤生成活性。本文旨在介绍*EML4-ALK*基因的结构、功能、生物学特征、检测方法及其在肺癌诊断治疗中的意义。

## Abstract (en)

It was only 3 years ago that the fusion gene between echinoderm microtubule-associated protein-like4 (EML4) and anaplastic lymphoma kinase (ALK) has been identified in a subset of non-small cell lung cancer (NSCLC). EML4-ALK is most often detected in never smokers with lung adenocarcinoma and has unique pathologic features. *EML4-ALK* fusion gene is oncogenic, which could be suppressed by ALK-inhibitor through blocking the downstream signaling passway of EML4-ALK. This review will focus on the molecular structure, function, biology, detection method and the diagnostic and therapeutic meaning of EML4-ALK of lung cancer.

肺癌是世界上最常见的恶性肿瘤之一，其中非小细胞肺癌（non-small cell lung cancer, NSCLC）约占80%。2007年Soda等^[1]^在NSCLC患者肿瘤标本中首次发现由2号染色体短臂内转位inv（2）（p21p23）形成的棘皮动物微管相关类蛋白4（echinoderm microtubule-associated protein-like4, EML4）与间变性淋巴瘤激酶（anaplastic lymphoma kinase, ALK）的融合基因。已有研究^[2]^表明EML4-ALK可诱导肿瘤生成，给予ALK抑制剂后肿瘤可迅速消退。在目前已报道的研究中*EML4-ALK*融合基因在NSCLC中的阳性率约为5%，*EML4-ALK*融合基因已成为肺癌临床治疗新靶点。本文旨在介绍*EML4-ALK*基因的结构、功能及生物学特征，探讨EML4-ALK的最佳检测方法，并评价其在肺癌治疗中的地位和意义。

## 1 *EML4-ALK*融合基因的结构与融合型

ALK属于胰岛素受体超家族，能与多种基因发生融合，如*NPM*-、*TPM3*-、*TFG*-、*ATIC*-、*CLTC*-等。它有着受体酪氨酸激酶的经典结构：细胞外配体结合区、跨膜区及细胞内酪氨酸激酶区。其细胞外区包含特殊的结合域：N端信号肽，2个甲基多巴，A5蛋白和受体蛋白酪氨酸磷酸酶μ（MAM, meprin, A5 protein and receptor protein ty-rosine phhosphatase mu）域，1个低密度脂蛋白类A（LDLa, Low-density lipoprotein A）基序以及1个靠近细胞膜的甘氨酸富集区（G-rich, Glycine-rich）。MAM域和G-rich区可能与ALK活化有关^[3]^。人类的ALK激酶区YxxxYY基序的第一个酪氨酸残基-Tyr1604已被证明与ALK激酶区的自体活化有关^[4]^。

EML4属于棘皮动物微管相关类蛋白家族，由N端Basic区、HELP域、WD-重复区构成。这3个区域都与EML4-ALK的肿瘤生成潜能有关，其中Basic区在EML4-ALK二聚体化过程中扮演重要角色，去除Basic区可使EML4-ALK融合蛋白的催化活性大大减低（84%），因此，Basic区可能为EML4-ALK融合蛋白产生效应的关键区域。Basic区靠近N端包含有一段螺旋卷曲，全部由EML4的2号外显子编码，称为CC（coiled coil螺旋卷曲）区，在多种ALK融合基因的融合对象中都存在，它可能是Ba-sic区内参与EML4-ALK二聚体化的主要基序^[1, 5]^。

EML4与ALK的基因序列在2号染色体短臂上方向相反，相隔12 Mb，融合时须二者之一反向与对方相接，二者断裂并相接的位置是区分各种*EML4-ALK*基因融合型的依据。所有*ALK*基因融合都发生在20号外显子编码的一段序列，而EML4断裂点则表现出多变性，已经检测到的断裂点有2、6、13、14、15、17、18、20号外显子，形成了11种*EML4-ALK*融合基因型，它们中的大部分被证实有促进肿瘤生成的活性^[1, 5-11]^。

## 2 *EML4-ALK*融合基因的信号传导通路及相关基因

*NPM-ALK*是目前研究最多的一种ALK融合基因，它的信号传导主要和PLCγ、PI3K/Akt、Ras/Mek/Erk及JAK3/STAT3通路有关。Li等^[12]^采用小分子ALK抑制剂阻断*EML4-ALK*融合基因的信号传导通路，探索其下游的信号传导，结果发现EML4-ALK与NPM-ALK有相似的下游信号传导通路，主要包括Akt、Erk和STAT3（图 1）。

**1:**
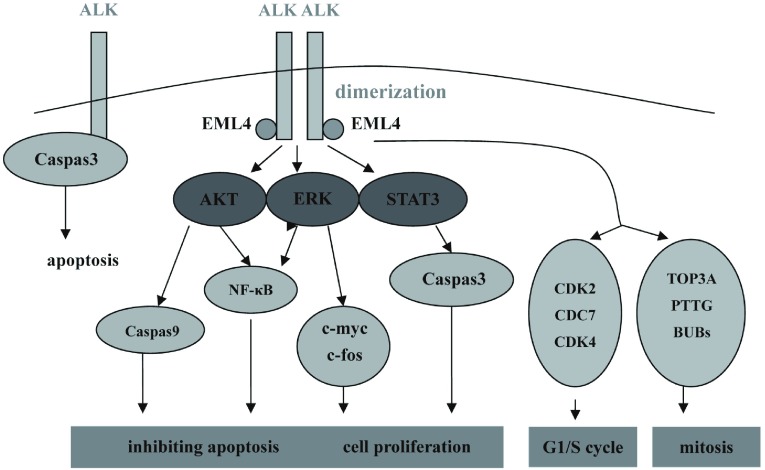
*EML4-ALK*融合基因和重要的下游信号传导通路 ALK fusion oncogenes and important downstream signaling pathways

与Li等^[12]^的研究结论不同，Koivunen等^[7]^发现ALK特异性抑制剂TAE684治疗与H3122细胞凋亡以及Akt和Erk1/2信号的下调相关，但却仅引起了H3122细胞STAT3磷酸化水平很小程度上的降低。Choi等^[6]^的研究中也有类似发现，转染了EML4-ALK的HEK293细胞在表现出肿瘤生成活性的同时，活化了*Fos*和*Myc*基因的启动子，诱导了NF-κB结合序列的活化，但*STAT3*基因没有明显变化。而最近Takezawa等^[13]^报道ALK抑制剂诱导的EML4- ALK阳性肺癌细胞的凋亡主要是由BIM上调抑制Erk通路以及survivin下调抑制STAT3通路介导。

STAT3通路对于NPM-ALK介导的淋巴瘤生成十分重要，单独抑制STAT3即能充分诱导细胞凋亡。以上关于*EML4-ALK*融合基因的研究中STAT3信号通路的作用变化不一，可能的原因是：①表达*EML4-ALK*融合基因的不同细胞系对EML4-ALK信号传导通路的依赖性存在差异。Koivunen等^[7]^发现转染了*EML4-ALK*融合基因的细胞系H3122在体内和体外试验中都对TAE684十分敏感，而另外2种表达*EML4-ALK*融合基因的细胞系，一种（H2228）对TAE684产生耐药，而另一种（DFCI032）则需要同时使用EGFR和ERBB2抑制剂才能抑制细胞生长；②表达*EML4-ALK*融合基因的不同细胞系其ALK信号传导的侧重点可能不同。Li等^[12]^发现转染了*EML4-ALK*融合基因的H2228细胞在经过TAE684治疗后出现了细胞周期阻滞和细胞凋亡，与细胞周期调控相关的基因*CDC2*、*CDC7*、*CDK4*等都下调了，ALK、Akt、STAT3、ERK的磷酸化也受到抑制；而转染了*EML4-ALK*融合基因的H3122细胞经TAE684作用后则侧重于促进细胞凋亡，对细胞周期的阻滞作用不明显。因此与细胞凋亡和存活相关的基因*STAT3*及*Akt*均表现出磷酸化水平明显减低，而与细胞生长及分化相关的基因ERK变化则不明显。

## 3 *EML4-ALK*融合基因阳性NSCLC患者的临床、病理及生物学特征

目前研究^[14]^发现年龄 < 50岁的患者较年龄≥50岁的患者*EML4-ALK*融合基因阳性率高（*P* < 0.01）。吸烟者较不吸烟者的阳性率低（*P* < 0.000, 1）^[15]^。Inamura等^[16]^在2008年的研究结果中显示肿瘤直径大小与*EML4-ALK*融合基因表达无关，但在2009年扩大样本量的研究^[14]^中发现肿瘤直径 < 30 mm较≥30 mm的患者*EML4-ALK*融合基因阳性率高（*P* < 0.05）。目前尚未发现性别、分期与*EML4-ALK*融合基因表达相关。

*EML4-ALK*融合基因在腺癌中的阳性率高于非腺癌（*P* < 0.05）^[10, 17]^，腺泡癌的阳性率高于其它类型的腺癌（*P* < 0.001）^[14, 16]^。与*EML4-ALK*融合基因阴性者相比，*EML4-ALK*融合基因阳性肺癌组织的癌细胞内、外富含粘液素（*P* < 0.000, 1），由于过多的细胞外粘液素，肺癌组织形态呈筛孔样（*P* < 0.000, 1），细胞内富含粘液素则形成印戒细胞^[18]^，印戒细胞的比例超过总细胞数的10%^[19]^。Takahashi等^[10]^报道分化差的较分化好的肺癌*EML4-ALK*融合基因阳性率高（*P* < 0.01），而其它研究中未发现组织分化程度与*EML4-ALK*融合基因阳性率相关。

存在*EGFR*、*K-Ras*基因突变的患者，其*EML4-ALK*融合基因阳性率较*EGFR*及*K-Ras*基因野生型者低（*P* < 0.05）（表 1）。多数文献报道*EML4-ALK*融合基因阳性的肺癌患者，通常不伴有*EGFR*和/或*K-Ras*基因突变。

**1:** 肺腺癌中*EML4-ALK*融合基因阳性与*EGFR*、*K-ras*基因突变的关系 Relationship between EML4-ALK fusion and genetic features in lung adenocarcinomas

Refercence	EGFR mutation			EGFR wildtype		*P*	K-Ras mutation			K-Ras wildtype		*P*
	*n*	EML4-ALK(+)		*n*	EML4-ALK(+)		*n*	EML4-ALK(+)		*n*	EML4-ALK(+)	
Inamura^[16]^	41	0		33	5	0.034	7	0		24	5	0.92
lnamura^[14]^	41	0		39	11	<0.001	7	0		61	11	0.49
Wong^[8]^	125	0		141	13	0.001	22	0		244	13	0.61
Takahashi^[10]^	105	0		106	5	0.072	29	0		182	5	0.81

## 4 *EML4-ALK*融合基因的检测方法评价

目前研究报道的肺癌组织中*EML4-ALK*融合基因检测的阳性率有很大差别，除了病例选择上的偏倚外，检测方法的不统一、无标准也是主要相关原因。常用的检测*EML4-ALK*融合基因的方法主要有反转录-聚合酶链反应（reverse transcription-polymerase chain reaction, RT-PCR）法、荧光原位杂交（ffuorescence in situ hybridization, FISH）法和免疫组织化学（immunohistochemical, IHC）法。

RT-PCR法是一种敏感性高的基因检测方法，多重RT-PCR常用来鉴别*EML4-ALK*基因的融合型。但RT-PCR需要高质量的RNA，石蜡切片中的DNA及RNA在检测过程中会逐渐降解，影响检测结果的准确性，因此，RT-PCR法更适用于新鲜标本的基因检测。

FISH法采用探针特异性标记细胞核中ALK断裂点来检测ALK重排，分离的荧光信号提示存在ALK重排。当IHC结果为弱阳性或阳性部位十分有限的时候，可用FISH法进行确认。但由于其价格昂贵，分离的荧光信号不易解释，FISH还不适合成为*EML4-ALK*融合基因的筛查手段。

IHC法是实验室最常用的蛋白筛查与诊断方法，操作简便，且已有研究表明IHC检测*EML4-ALK*融合基因的结果和RT-PCR、FISH法的检测结果具有一致性，建议使用IHC法作为筛查*EML4-ALK*融合基因的方法，但IHC法的敏感性不如RT-PCR和FISH法。有研究^[9, 20]^表明使用抗体增强剂（iAEP）及敏感性、特异性高的抗体，如5A4抗体、CD246抗体（克隆号：D5F3）可以克服这一缺点，使得IHC法用于筛选ALK抑制剂的潜在有效人群成为可能。

## 5 *EML4-ALK*融合基因阳性NSCLC患者的预后、临床过程及对治疗的反应

从发现*EML4-ALK*融合基因在肺癌组织中表达至今仅3年，很多研究者已把*EML4-ALK*融合基因阳性的肺癌患者看做独立的肺癌亚型，但对于这部分患者的临床结局及对治疗的反应尚没有大宗的病例报告，现在仅能从个别研究中窥见一斑。Takahashi等^[10]^观察了4例*EML4-ALK*融合基因阳性的可手术肺癌患者的预后及临床病程：其中2例Ⅰa期的腺癌患者生存期超过了60个月，没有出现复发和转移；1例Ⅲa期患者生存超过99个月，在手术后73个月出现肺转移；另1例Ⅲa期患者在术后8个月出现脑转移，至研究结束已存活53个月。Murakamia等^[21]^曾报道了1例EML4-ALK阳性患者手术后20年复发。以上研究提示*EML4-ALK*融合基因阳性的NSCLC患者可能临床病程较长，预后较好，但尚需更多病例的观察与比较才能确认。

有研究^[22]^发现*EML4-ALK*融合基因阳性患者及EGFR、*EML4-ALK*基因双野生型（WT/WT）的患者对酪氨酸激酶抑制剂（tyrosine kinase inhibitor, TKI）治疗的反应不明显，对以铂类为基础的联合化疗的反应率也低于*EGFR*突变患者，提示*EML4-ALK*融合基因阳性患者不同于*EGFR*突变患者，似乎不能作为化疗疗效的正面预测因子。此外，*EML4-ALK*融合基因阳性患者的中位生存期（median survival time, MST）及总生存期（overall survival, OS）较WT/WT患者长一些但无统计学差异，*EGFR*基因突变患者的疾病进展时间（time to progression, TP）及总生存时间（overall survival, OS）则明显长于WT/WT者。由以上结果发现尽管*EML4-ALK*融合基因阳性患者对治疗的反应率及生存指标方面都略逊于*EGFR*突变患者，但好于WT/WT患者，给予相应的抑制剂治疗后，*EML4-ALK*融合基因阳性患者的生存期可能会更长。

一项关于ALK抑制剂TAE684的研究^[7]^报道，3个存在EML4-ALK易位的细胞系中H3122细胞系对TAE684治疗特别敏感；DFCI032细胞系中同时存在*EGFR*和*ERBB2*基因的共活化，对TAE684治疗不十分敏感；而H2228细胞系则对TAE684产生耐药，未检测到其它激酶的共活化，耐药原因尚不清楚。Choi等^[23]^在1例ALK抑制剂耐药的NSCLC患者的瘤细胞中检测到了*EML4-ALK*融合基因内的二次突变，分别发生在第4374号碱基（G→A）和第4493号碱基（C→A），导致对应位置的1156号氨基酸（半胱氨酸→酪氨酸）和1196号氨基酸的改变（亮氨酸→甲硫氨酸），并证实这两个点突变都与ALK抑制剂耐药有关，但不清楚这两个点突变发生在用药前还是用药后。以上研究表明，EML4-ALK抑制剂在临床上可能只对一部分包含EML4-ALK易位的NSCLC患者有效，其它受体酪氨酸激酶的活化以及*EML4-ALK*融合基因内的二次突变可能是引起ALK抑制剂耐药的原因。

Soda等^[2]^培育了肺泡上皮细胞表达*EML4-ALK*融合基因的转基因小鼠，几周后发现这些小鼠的双肺长出了成百上千的腺癌结节，给予ALK活性抑制剂（2, 4-嘧啶二胺）后，这些癌结节很快消退，但残存的肿瘤细胞仍有活性，停药25天后小鼠肺部又长出了大小不一的癌结节。这些结果支持EML4-ALK是驱动肺癌发生的原癌基因的理论，为使ALK抑制剂达到更好更稳定的疗效，需要延长给药时间或加大给药剂量。另外，转染*EML4-ALK*融合基因后的细胞中可能有其它信号通路的激活，通过抑制其它激活的信号通路也是提高疗效的一种潜在方法。

目前已进入临床研究的ALK抑制剂只有crizotinib（PF-02341066），它同时也是MET抑制剂。Ⅰ期临床试验^[24]^显示其客观缓解率为57%，疾病控制率是90%，中位无进展生存期9.2个月。Ⅱ期临床试验^[25]^中客观缓解率为64%，疾病控制率为90%，生存终点还在观察中。不良反应主要是胃肠道反应，包括恶心、呕吐和腹泻。该药抗癌效果满意，安全性好，已进入Ⅲ期临床试验（Pro-fle-1007）。

## 6 结论

综上所述，*EML4-ALK*融合基因已经成为NSCLC治疗的新靶点，一部分EML4-ALK阳性的患者可从ALK抑制剂治疗中获益。*EML4-ALK*融合基因阳性的患者，通常为年龄 < 50岁、不吸烟、肿瘤直径 < 30 mm、病理类型为腺癌，不伴有*EGFR*、*K-ras*基因突变的个体。在临床研究中可先通过以上特征初步筛选出可能的获益人群，之后再行*EML4-ALK*融合基因或蛋白检测可事半功倍，IHC法可能是目前筛查*EML4-ALK*融合基因的最佳手段。

但是，*EML4-ALK*融合基因的研究仍有许多待解决的问题，比如Martelli等^[26]^的研究中使用RT-PCR法在120例冰冻NSCLC标本及远离肿瘤的肺组织中均检测到了*EML4-ALK*融合基因，但未检测到EML4-ALK蛋白表达，研究者认为这是因为表达*EML4-ALK*融合基因的细胞数太少所致。已往大多数研究已从基因和蛋白水平证实了肺癌中*EML4-ALK*融合基因存在，且基因和蛋白检测结果一致。该实验中基因蛋白分离的情况值得深思，蛋白水平检测阴性的情况下，尽管检测到了*EML4-ALK*融合基因，该基因是否行使了促进肿瘤生成的功能，这类患者是否适合接受ALK抑制剂治疗有待继续深入研究。另外，EML4-ALK的信号通路方面的研究较少，该信号通路是否和EGFR信号通路有相互交叠及相互影响尚不明确，需要进一步研究探讨。
